# Assessing *Gammapapillomavirus* infections of mucosal epithelia with two broad-spectrum PCR protocols

**DOI:** 10.1186/s12879-020-4893-3

**Published:** 2020-04-07

**Authors:** Elisa M. Bolatti, Lea Hošnjak, Diego Chouhy, Pablo E. Casal, María F. Re-Louhau, Hebe Bottai, Kristina Fujs Komloš, Mario Poljak, Adriana A. Giri

**Affiliations:** 1 Grupo Virología Humana, Instituto de Biología Molecular y Celular de Rosario (CONICET), Suipacha 590, 2000 Rosario, Argentina; 2 Área Virología, Facultad de Ciencias Bioquímicas y Farmacéuticas, Universidad Nacional de Rosario, Suipacha 531, 2000 Rosario, Argentina; 3 Faculty of Medicine, Institute of Microbiology and Immunology, University of Ljubljana, Zaloška 4, 1000 Ljubljana, Slovenia; 4 Área Estadística y Procesamiento de Datos, Facultad de Ciencias Bioquímicas y Farmacéuticas, Universidad Nacional de Rosario, Suipacha 531, 2000 Rosario, Argentina

**Keywords:** *Gammapapillomavirus*, Mucosal epithelia, *Gamma*-PV/CUT PCR assays, Anal canal, Prevalence, Persistence

## Abstract

**Background:**

Human papillomaviruses (HPVs) have been divided into mucosal and cutaneous types according to their primary epithelial tissue tropism. However, recent studies showed the presence of several cutaneous types in mucosal lesions and healthy mucosa from different anatomical sites.

**Methods:**

Here, the HPV prevalence and type-specific distribution were assessed in a variety of mucosal samples from 435 individuals using a combination of two established broad-spectrum primer systems: *Gamma*-PV PCR and CUT PCR.

**Results:**

Overall HPV prevalence in anal canal swabs, cervical cancer biopsies, genital warts and oral swabs was 85, 47, 62 and 4%, respectively. In anal canal swabs, *Alpha*-PVs were most frequently found (59%), followed by *Gamma*- (37%) and *Beta*-PVs (4%). The prevalence and persistence of HPV infection in the anal canal of 226 individuals were further explored. Overall HPV, *Gamma*-PVs and multiple HPV infections were significantly higher in men vs. women (*p* = 0.034, *p* = 0.027 and *p* = 0.003, respectively); multiple HPV infections were more common in individuals ≤40 years (*p* = 0.05), and significantly higher prevalence of *Gamma*-PVs and multiple HPV infections was observed in HIV-1-positive vs. HIV-1-negative individuals (*p* = 0.003 and *p* = 0.04, respectively). Out of 21 patients with follow-up anal swabs, only one persistent infection with the same type (HPV58) was detected.

**Conclusions:**

Our findings suggest that *Gamma*-PVs (except species *Gamma*-6) are ubiquitous viruses with dual muco-cutaneous tissue tropism. Anal canal *Gamma*-PV infections may be associated with sexual behavior and the host immune status. This study expands the knowledge on *Gamma*-PVs’ tissue tropism, providing valuable data on the characteristics of HPV infection in the anal canal.

## Background

To date, more than 320 different human papillomavirus (HPV) types have been identified according to phylogenetic relationships of their complete L1 gene sequences, within five genera of the *Papillomaviridae* family (*Alpha-*, *Beta-*, *Gamma-*, *Mu-* and *Nu-*PV) [1–3]. Based on the epithelial tissue tropism, HPVs have been originally subdivided into mucosal and cutaneous types [1]. Mucosal HPV types, typically clustering to the *Alpha*-PV genus, which also contains several predominantly cutaneous HPV types, are associated with the development of pre-malignant and malignant lesions of the anogenital, oral and oropharyngeal epithelia [4]. *Alpha*-PV infections of the anal canal have frequently been detected in human immunodeficiency virus (HIV-1)-infected men who have sex with men (MSM) [5, 6]. Moreover, HIV-1-positive MSM have approximately 60 times higher risk of developing anal cancer than the general population [7, 8], and also show higher risk with respect to men who have sex with women (MSW) [9], women [10], and HIV-1-negative MSM [11], but in lower proportions.

Cutaneous HPVs are dispersed across all five HPV genera and have most frequently been detected in healthy skin samples, suggesting their commensal nature [2, 12]. However, several studies showed a high prevalence of *Beta*- and *Gamma*-PVs at several anatomical sites, different from sites in which they were originally identified, including cutaneous and mucosal lesions and healthy mucosa, suggesting their double, muco-cutaneous tissue tropism, and adding more questions about their clinical importance [13–15]. In contrast to *Beta*-PVs where the number of studies supporting their oncogenic potential in the development of skin cancer has increased over time [16], the role of *Gamma*-PVs in the development of malignant mucosal/cutaneous lesions has been poorly described [17–19]. Interestingly, our recent study, together with previous data, suggests a potential active role of *Gamma*-PVs in the development of pre-malignant skin lesions in immunocompetent individuals [20, 21]. On the other hand, patients with a rare inherited immunodeficiency have been found to be uniquely susceptible to *Gamma*-PV-associated skin warts [22]. Furthermore, some *Gamma*-PV types, especially those belonging to the *Gamma*-6 species, have been detected in *Alpha*-PV-negative anogenital warts [23, 24] and cervical precancerous lesions [13, 17, 18, 25, 26].

Even though the presence of different HPV types has recently been demonstrated in the anal canal of men, the used methodologies only enabled the identification of a limited number of *Alpha*-, *Beta*- and/or *Gamma*-PV types [5, 10, 27, 28]. Moreover, very little is known about the natural history of infection with predominantly cutaneous HPVs in the anogenital region [27].

The objective of the present study was to assess the prevalence and type-specific distribution of a wide range of HPV types in a variety of mucosal samples, using a combination of two established broad-spectrum primer systems, enhancing the ability to detect *Gamma*-PVs. Additionally, the prevalence and persistence of HPV infection in the anal canal of HIV-1-positive and -negative men and women were comparatively evaluated.

## Methods

### Patients’ data, sample collection and processing

Samples of total DNA (*n* = 458), extracted from 249 swabs of the anal canal (226 initial and 23 follow-up samples), 94 cervical cancer formalin-fixed paraffin-embedded (FFPE) tissue samples, 21 genital warts (11 swabs and 10 biopsies) from different anatomical sites [foreskin (*n* = 4), scrotum (*n* = 2), penile glans (*n* = 2), perianal area (*n* = 4) and pubis (*n* = 9)], and 94 oral swabs collected during routine paternity testing, were obtained from the archival collection of samples of the Instituto de Biología Molecular y Celular de Rosario (Rosario, Argentina) and the Institute of Microbiology and Immunology, Faculty of Medicine, University of Ljubljana (Ljubljana, Slovenia). Before subsequent analyses, all samples were collected, processed, and stored at − 80 °C, as described previously [11, 27, 29–32]. The adequacy of samples for downstream analyses was determined by PCR amplification of the human beta-globin gene, as previously described [33].

In more detail, and in order to compare the prevalence and persistence of HPV infection in the anal canal of HIV-positive and HIV-negative subjects, samples and data from 226 patients were mostly obtained from our previous studies on *Alpha*- and *Beta*-PV infections in the anal canal [11, 27]. Additionally, 23 follow-up samples from eligible individuals that were obtained after the conclusion of two previous studies were included in the present study. Subjects were 18 to 66 years old (median age = 33 years) and 20/226 (9%) represented HIV-1 positive men. As all anal canal swab samples were collected during standard proctologic exams unfortunately information on the presence of specific anal lesions could not be obtained for all patients. For the 133 patients for whom clinical data were available, more than two-thirds (90/133; 67.6%) had a clinically evident HPV-related or HPV-unrelated anal pathology, including anal warts (77/133; 57.9%), hemorrhoids (5/133; 3.8%) and anal fissure (3/133; 2.3%), as reported in our previous study [27], suggesting that anal warts were most probably the predominant HPV-related pathology in our patient population. Additionally, all male participants were MSM and had a history of receptive anal sexual intercourse. Unfortunately, no data on the sexual behavior of women could be obtained. Moreover, a total of 21 subjects (20 males and one female), age range of 22 to 42 years (median age = 30 years), of which two men were HIV-1-positive, were enrolled in the current analysis of HPV persistence in the anal canal, with a follow-up anal swabs collected in period ranging from 2 to 82 months (average = 36 months; median = 43 months). At enrolment, 10/21 (47.6%) of these patients had anal warts, 4/21 (19.0%) had no clinically evident abnormalities, two patients had anal fissure and hemorrhoids, respectively, and no data were available for 5/21 (23.8%) patients (Table S2).

### Detection of HPV infection

The presence of HPV infection was determined using two well-established HPV generic primer systems, *Gamma*-PV PCR [21] and CUT PCR [30]. All PCR reactions were performed as described previously [21], using the following reaction controls: a negative control (5 ng of human placental DNA) to check for the reaction’s specificity, a reagent control (H_2_O instead of the sample) to check for carry-over contamination, and 100 copies of cloned HPV4 or HPV10 in a background of 5 ng human placental DNA as a positive control per PCR run. All pre- and post-PCR procedures were carried out in separate cabinets and rooms. Amplicons derived from CUT (≈370 bp) and *Gamma*-PV PCR systems (≈158 bp) were sequenced by Sanger methodology. The obtained nucleotide sequences were compared to available HPV-sequences in the GenBank database (https://www.ncbi.nlm.nih.gov/genbank/), using the Blast algorithm (https://blast.ncbi.nlm.nih.gov/Blast.cgi). Following CUT PCR, a novel putative HPV type was determined when the fragment sequence showed less than 90% nucleotide identity to L1 ORFs of any of the previously known HPV types [1]. The same criterion was applied to classify types/putative types derived from the *Gamma*-PV PCR, which is based on the amplification of partial HPV E1 sequences, due to the similar nucleotide identities obtained in a pairwise comparison analysis between L1 and E1 ORFs, as shown previously [21].

Additionally, in the analysis of HPV persistence in the anal canal, the obtained results were compared to the results of HPV typing using the commercially available Linear Array HPV Genotyping Test (Roche Diagnostics, Mannheim, Germany) and RHA Kit Skin (Beta) HPV assay (RHA; Diassay BV, Rijswijk, The Netherlands), as described previously [11, 27].

### Phylogenetic analysis of novel putative HPV types

Sequences of E1 gene regions of 166 representative HPV types from the *Gamma*-PV genus, available at http://www.nordicehealth.se/hpvcenter/ and Papillomavirus episteme (http://pave.niaid.nih.gov), were used as a database for the phylogenetic analysis of novel putative HPV types. Multiple sequence and pairwise alignments were constructed using the ClustalW algorithm of the MEGA6 software package [34] at the amino acid (aa) level. The alignment of novel putative HPV E1 sequences was obtained with MAFFT’s “Align fragment sequences to an MSA” tool [35]. The phylogenetic relationships between representative *Gamma*-PV types and novel putative HPV E1 sequences were inferred by Bayesian analysis using Beast version 1.7.5 [36]. To do so, Markov Chain Monte Carlo (MCMC) simulations were performed during 2 × 10^7^ generations, sampling one state every 1000 generations, with a burnin of 10%. The setting “Create tree log file with branch length in substitutions” was selected to obtain the phylogram log file. The evolutionary substitution model selected for each run was GTR + I + Γ. Statistical convergence of MCMC was assessed visually by the traceplot and by calculating the effective sample size using TRACER v1.4 (available at http://beast.bio.ed.ac.uk/Tracer). The maximum clade credibility tree across all the plausible trees generated by BEAST was then computed with the TreeAnnotator program available in the BEAST package.

### Statistical analysis

The statistical analysis of categorical variables was performed by Chi square and Fisher Exact tests. Multivariate analysis (logistic regression) was performed for all variables. *p* values below 0.05 were regarded as statistically significant.

### Nucleotide sequence accession number

The GenBank/EMBL/DDBJ accession numbers for the novel HPV putative types reported in this paper are: EP26 (MK510728), EP27 (MK510729), EP28 (MK510730), EP29 (MK510731).

## Results

### HPV detection and typing using Gamma-PV and CUT PCR systems

**Fig. 1:**
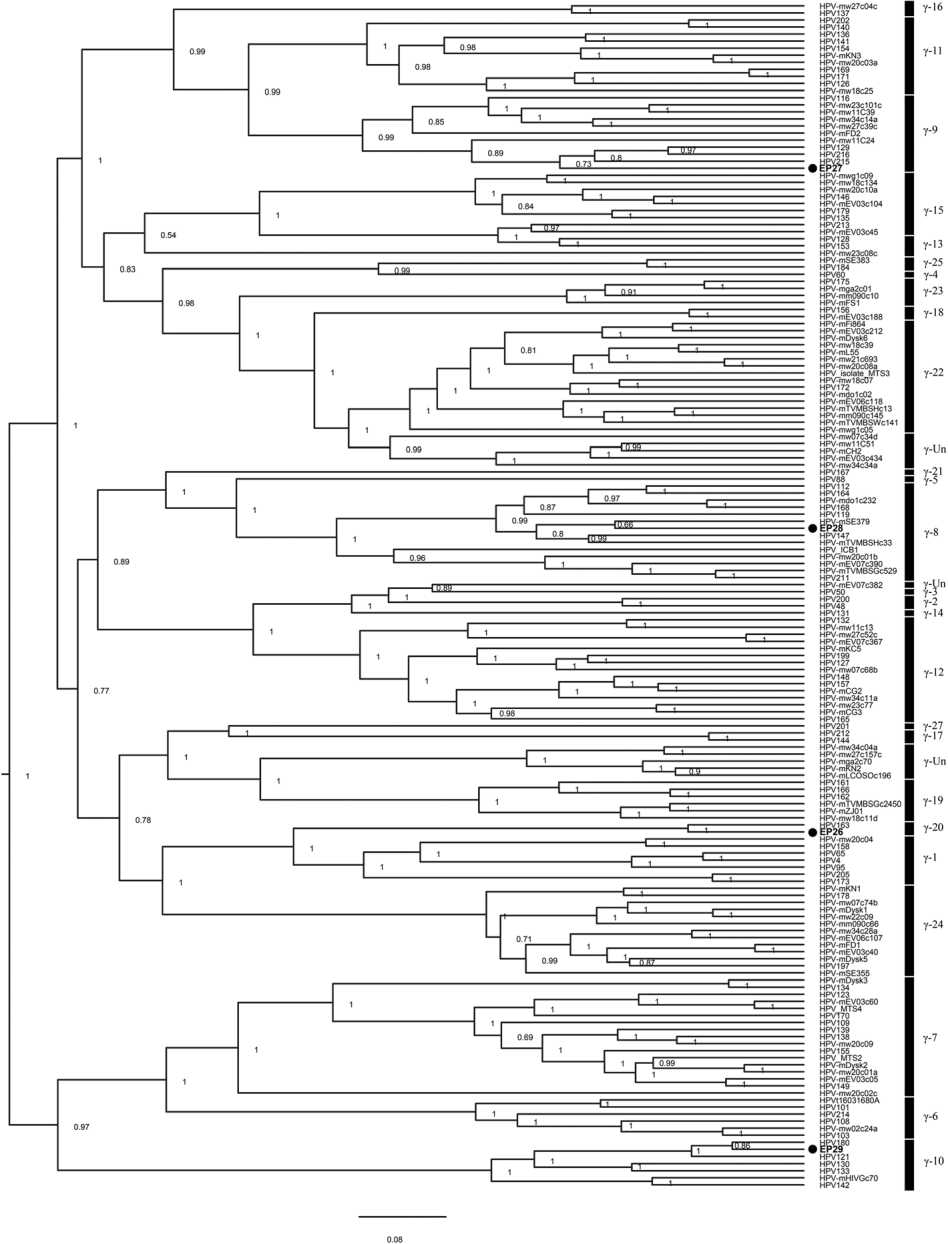
Phylogenetic tree of novel putative HPV types and 166 representative HPV sequences from *Gamma*-PV genus. Phylogenetic analysis of partial E1 nucleotide sequences obtained with *Gamma*-PV PCR (~ 158 bp) and the phylogenetic position of four novel putative HPV types (EP26, EP27, EP28, EP29) identified in this study. Only bayesian posterior probability (BPP) values of > 0.50 are shown. Novel putative HPV types identified in this work are indicated with solid dots. Some clades were collapsed to facilitate the visual analysis. The raw phylogenetic trees are available upon request

A total of 71 different HPV types/putative types (27 *Alpha*-, 7 *Beta*- and 37 *Gamma*-PV), clustering to 30 PV species (9 *Alpha*-, 3 *Beta*- and 18 *Gamma*-PV), were identified in HPV-positive mucosal samples using both broad-spectrum PCR protocols (Table 1, Table S1). It should be noted that only five HPV types (HPV38, HPV133, HPV161, HPV135 and HPV180) were consistently simultaneously detected in the same samples by both primer systems. In addition, four novel putative HPV types (EP26, EP27, EP28, EP29) were identified in this set of samples, all of them being found in anal canal swabs with the *Gamma*-PV PCR primer system (Table 1, Table S1). Phylogenetic relationships between these novel putative HPV types and sequences of E1 gene regions of 166 representative HPV types from the *Gamma*-PV genus are shown in Fig. 1. Putative HPV type EP26 clusters within the species *Gamma*-20 and shares a 90% E1 ORF nucleotide identity with HPV163. EP27 belongs to the species *Gamma*-9 and shows the highest nucleotide identity with HPV216 (88%). On the other hand, EP28 exhibits an 87% nucleotide identity with HPV-mSE379, clustering within the *Gamma*-8 species, while EP29 belongs to the *Gamma*-10 species, sharing a 90% nucleotide identity with HPV180.

**Table 1:** HPV detection in 458 samples of mucosal epithelia

	Patients (*n*)	Samples (*n*)	HPV-prevalence *n* (%)	No. of detected HPV types	No. of detected novel putative HPV types	No. of detected HPV species	Detected multiple infections *n* (%)
Anal canal swabs	226	249	192 (85)	67	4	29	105 (48)
Cervical cancer biopsies	94	94	44 (47)	6	0	3	7 (7)
Genital wart samples	21	21	13 (62)	3	0	3	1 (5)
Oral swabs	94	94	4 (4)	3	0	3	3 (3)
**Total**	**435**	**458**	**253 (55)**	**70**^a^	**4**	**30**^b^	**116 (25)**

^a^The following 9 HPV types were identified in different sets of samples: HPV6, HPV11 and HPV-EV07c385 (anal canal and genital warts); HPV16, HPV33, HPV45 and HPV58 (anal canal and cervical cancer); HPV18 (anal canal, cervical cancer and oral swabs); HPV38 (anal canal and oral swabs)

^b^The following PV species were identified in different sets of samples: *Alpha*-7 (anal canal, cervical cancer and oral swabs); *Alpha*-9 (anal canal and cervical cancer); *Alpha*-10 and *Gamma*-18 (anal canal and genital warts); *Beta*-2 (anal canal and oral swabs)

### HPV prevalence and genera distribution in the mucosal epithelia

Overall, HPV DNA was present in 55% (253/458) of all tested mucosal samples and HPV prevalence was determined at 58% (253/435) (Table 1). Specifically, HPV prevalence in anal canal swabs, cervical cancer biopsies, genital warts and oral swabs was estimated at 85% (192/226), 47% (44/94), 62% (13/21) and 4% (4/94), respectively. Additionally, 25% (116/458) of included samples contained multiple HPV infections, most frequently in swabs of the anal canal (105/226; 48%) (Table 1).

**Fig. 2:**
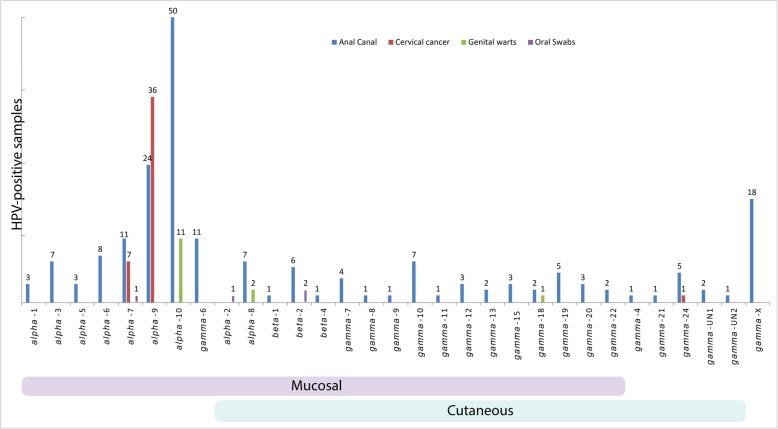
Detection of *Alpha*-, *Bet*a- and *Gamma*-PVs in 458 samples of mucosal epithelia. Color bars under X axis show HPV species according to cutaneous and mucosal tissue tropism [1]. HPV species over merged bars exhibit dual tissue tropism. *Gamma*-X refers to sequences grouped in the genus *Gamma*-PV from which the prevailing HPV types/s and species could not be determined

As shown in Fig. 2 and Table S1, in swabs of the anal canal members of the *Alpha*-PV genus were the most frequently detected (113/192; 59%), followed by *Gamma*- (71/192; 37%) and *Beta*-PV genera (8/192; 4%). *Alpha*-PV genus types were also the most frequently detected in cervical cancer biopsies (43/44; 98%) and genital wart samples (13/14; 93%). While HPV types clustering to the species *Alpha*-10 were the most frequent in anal canal swabs (50/192; 26%) and genital wart samples (11/14; 79%), HPVs grouped within the species *Alpha*-9 were most frequently identified in cervical cancer biopsies (36/44; 82%). The prevailing HPV type detected in both anal canal swabs (37/192; 19%) and genital wart samples (7/14; 50%) was HPV6 (*Alpha*-10) and, as expected, HPV16 (*Alpha*-9) was the most prevalent in cervical cancer biopsies (32/44; 73%). Nevertheless that a wide variety of *Gamma*-PVs, clustering to different *Gamma*-PV species, were detected in the anal canal (Fig. 2, Table S1), those grouped within the species *Gamma*-6 were the most frequently identified (11/192; 6%). It should be noted that unclear electropherograms from which the prevailing HPV type/s and species could not be determined (*Gamma*-X) were obtained from 18 *Gamma*-PV-positive anal canal swab samples (Fig. 2, Table S1). The frequency of HPV infection in oral swab samples was relatively low and the majority of positive samples contained *Alpha*-PVs [HPV10 (*Alpha*-2) and HPV18 (*Alpha*-7)] or *Beta*-PVs [HPV38 (*Beta*-2)] (Fig. 2, Table S1).

### HPV prevalence and persistence in samples of the anal canal

The overall prevalence of HPV infection in the anal canal of the 226 patients included in the study was estimated at 79% (Table 2). Particularly, *Alpha*-, *Beta*- and *Gamma*-PVs were present in 59, 3 and 35% of the initial samples obtained from all study participants at the first visit, respectively. In addition, multiple infections were detected in 43% of included samples.

**Table 2:** Prevalence of overall HPV, *Alpha*-*, Beta*- and *Gamma*-PV infections in the anal canal of 226 individuals

Variable	No. of patients	All HPVs *n* (%)	*p*	*Alpha*-PVs *n* (%)	*p*	*Beta*-PVs *n* (%)	*p*	*Gamma*-PVs *n* (%)	*p*	Multiple infections *n* (%)	*p*
All Subjects	226	179 (79)		133 (59)		7 (3)		78 (35)		98 (43)	
Gender											
Female	51	35 (69)	**0.034**	28 (55)	0.515	1 (2)	0.59	11 (22)	**0.027**	13 (26)	**0.003**
Male (MSM)	175	144 (82)		105 (60)		6 (3)		67 (38)		85 (49)	
Age											
18-30 years	97	76 (78)	0.69	59 (61)	0.86	4 (4)	0.40	32 (33)	0.17	44 (45)	
31-40 years	88	72 (82)		50 (56)		1 (1)		36 (40)		43 (49)	**0.05**
41-66 years	41	31 (76)		24 (59)		2 (5)		10 (24)		11 (27)	
HIV-1 status											
HIV-1-positive	20	18 (90)	0.21	14 (70)	0.29	2 (7)	0.103	13 (65)	**0.003**	13 (65)	**0.04**
HIV-1-negative	206	161 (78)		119 (58)		5 (3)		65 (32)		85 (41)	

*p* values ≤ are depicted in bold

In comparison to women, the prevalence of overall HPV, *Gamma*-PV and multiple HPV infections was significantly higher in anal canal swab samples of men (82% vs. 69%, *p* = 0.034; 38% vs. 22%, *p* = 0.027 and 49% vs. 26%, *p* = 0.003, respectively) (Table 2). Multiple HPV infections were slightly more common among 18-30 and 31-40-year-old patients (45 and 49%, respectively), in comparison to individuals older than 40 years (27%) (*p* = 0.05). A significantly higher prevalence of *Gamma*-PVs and multiple HPV infections was observed in HIV-1-positive vs. HIV-1-negative individuals (65% vs. 32%, *p* = 0.003 and 65% vs. 41%, *p* = 0.04, respectively). All differences between gender, age and HIV-1 status observed during univariate analyses were additionally confirmed using multivariate analyses (Table 3). Thus, men had a higher risk for overall HPV infections, *Gamma*-PV infections and multiple HPV infections than women, and HIV-1-positive subjects had a higher risk for infections with *Gamma*-PV and for multiple HPV infections than HIV-1-negative individuals.

**Table 3:** Multivariate Analysis of variables associated with HPV infection in the anal canal of 226 individuals

Variable	HPV infection			*Gamma*-PV infection			Multiple HPV infection		
	*n* (%)	*p*	Odds Ratio [95% CI]	*n* (%)	*p*	Odds Ratio [95% CI]	*n* (%)	*p*	Odds Ratio [95% CI]
Gender									
Female	35 (69)	0.0369	2.12 [1.05–4.31]	11 (22)	0.048	2.25 [1.08–4.69]	13 (26)	0.023	2.29 [1.12-4.67]
Male (MSM)	144 (82)			67 (38)			85 (49)		
HIV status									
HIV-1-positive	18 (90)	NS	–	13 (65)	0.0124	3.47 [1. 31-9.22]	13 (65)	0.049	2.38 [1.01-6.37]
HIV-1-negative	161 (78)			65 (32)			85 (41)		

*NS* not significant

As shown in Table S2, out of 21 patients with follow-up anal swabs, persistent infection with the same HPV type was detected only in a single patient (HPV58, patient No.18; Table S2). Additionally, two individuals were HPV-negative throughout the follow-up period (patient No. 8 and No. 21; Table S2). A total of 52% (11/21) individuals were infected with different HPV types/putative types in the initial and follow-up anal samples, and 29% (6/21) had HPV-positive initial samples but tested negative in the follow-up samples. Only one patient tested HPV-positive in follow-up sample, while being previously HPV-negative (patient No. 2; Table S2).

## Discussion

With the exception of *Alpha*-PVs, which are etiologically associated with the development of more than 99% of cases of cervical cancer, 70–90% of cases of anal and vaginal cancers, 40% of cases of vulvar cancer, 47% of cases of penile cancer, 25-30% of cases of oropharyngeal cancer, and more than 90% of cases of genital warts and laryngeal papillomas [37, 38], knowledge is limited concerning prevalence and clinical importance of other HPV genera in the mucosal epithelia. The present analysis of 458 mucosal samples, including biopsies of genital warts and cervical cancer as well as anal and oral swabs, enabled the identification of a large number of HPV types, clustering to a diverse range of species of *Alpha*-, *Beta*- and *Gamma*-PV genera. Interestingly, only five HPV types were simultaneously detected by *Gamma*-PV and CUT PCR assays, showing the differential capacities of both primer systems on detecting diverse HPV types, as indicated previously [21]. Nevertheless that one set of broad-spectrum primers targeting the E1 gene has been described previously [39], the mentioned CP primers can mostly detect *Alpha*- and *Beta*-PV types and have different specificities than those used in our *Gamma*-PV assay (Table S3).

Thirty-six different *Gamma*-PVs, clustering into 18 species, with two of them described for the first time and not yet officially recognized (*Gamma*-Un1, *Gamma*-Un2; Fig. 2 and Table S1), were identified in the present study. Among all *Gamma*-PV-positive samples, members of the *Gamma*-6 species were the most frequently detected (11/73; 15%). Interestingly, none of them were identified in a variety of 653 cutaneous samples with the same testing approach [21]. These results, together with previously published findings [13, 17, 26, 28, 40–43], suggest the possible adaptation of the mentioned HPV types to the mucosal epithelium [44].

Our observations are not surprising since previous studies have demonstrated that typically cutaneous HPV types (*Beta*- and *Gamma*-PVs) were relatively common in the mucosal epithelia, suggesting a possible dual tissue tropism of the majority of HPV species [1, 13, 32, 28, 45, 46]. In addition, two *Gamma*-PV types were identified in a surface swab of a genital wart and a cervical cancer biopsy sample, possible as part of the normal mucosal microbiota, as reported previously [23, 26].

Although using CUT primers several high- and low-risk *Alpha*-PV types were identified in cervical cancer biopsies, a significantly lower HPV prevalence was found in the present study in comparison with previously published data [4]. The mentioned discrepancy could be a result of the fact that CUT primers were designed to detect the so-called “cutaneous” HPV types that are distributed across all five HPV genera [30]. Although CUT primers are able to detect mucosal types, their sensitivity for detection of *Alpha*-PV is much lower in comparison to other approaches using well-known *Alpha*-PV PCR primers, such as MY09/11, GP5+/6+ or SPF10, as previously reported [47]. Therefore, the CUT primers are not recommended for detection of clinically most relevant mucosal *Alpha*-PV HPV types associated with the development of several anogenital neoplasms, including cervical cancer. Instead, CUT primers can be considered as an additional tool for epidemiological studies, in combination with other testing approaches, such as the *Gamma*-PV PCR assay and/or with standard *Alpha*-PV primers, to explore the presence and HPV type diversity. Additionally, it should be considered that amplification of DNA sequences that are approximately 370 bp long could be compromised in FFPE tissue samples, leading to an underestimation of HPV prevalence.

In line with previous epidemiological studies that have analyzed the prevalence of HPV infection in anal canal of MSM [5, 27, 28], MSW [10] and women [9], in the present study significantly higher overall HPV prevalence and rate of multiple HPV infections were detected in swabs of the anal canal of MSM in comparison to women (*p* = 0.034 and *p* = 0.003, respectively). Nevertheless that the prevalence of *Alpha*-PV infections was similar in anal canal of MSM and women (*p* = 0.515), interestingly, the prevalence of *Gamma*-PVs was significantly higher among MSM (*p* = 0.027). Although the mentioned differences may have originated from the use of different HPV detection methods in different studies, it is likely that receptive anal sexual intercourse might indeed have resulted from more frequent acquisition of *Gamma*-PVs, as suggested previously for *Alpha*- and *Beta*-PVs [27, 48, 49]. On the other hand, it should be considered that anal HPV infections, especially with *Beta*- and *Gamma*-PVs, may occur through self- or partner- inoculation [50, 51].

In line with previous observations that younger age could be associated with the higher risk for acquiring anal HPV infection in men [10, 52], in the present study significantly higher prevalence of multiple HPV infections was detected in subjects that were 18-30 and 31-40 years old at the time of the study in comparison to older individuals (41-66 years) (*p* = 0.05). The age-specific HPV infection prevalence trends in the anogenital region may have resulted from younger subjects being more sexually active than older individuals, with up to 3-fold higher number of sexual partners [52].

Since *Beta*- and *Gamma*-PV prevalence data among HIV-1-positive individuals are conflicting [5, 27, 28, 41], HPV prevalence according to patients’ HIV-1 infection status was further investigated in the present study. In concordance with former reports [28, 41], the prevalence of *Gamma*-PVs and multiple HPV infections was significantly higher in HIV-1-positive subjects in comparison to HIV-1-negative individuals (*p* = 0.003 and *p* = 0.04, respectively). As the contradictory results obtained in epidemiological studies could be attributed to differences in the immune status of HIV-1-infected individuals and to the heterogeneity of enrolled patients and HPV detection methods used, additional studies are warranted to further explore the association(s) between HIV-1 and anal HPV infection.

Due to the scarce knowledge on the natural history of non-*Alpha-*PV infection in the anal canal [27], 21 patients were prospectively followed-up in our study. While all *Beta*- and *Gamma*-PV infections were found to be transient, a persistent *Alpha*-PV infection was detected in one patient. Although transient *Gamma*-PV infections have previously been described in healthy skin samples [53], in studies using reverse-line blot hybridization techniques, it has been demonstrated that *Alpha*- and *Beta*-PVs can establish persistent infections of the anal canal [11, 27]. It should be noted that in the present study, HPV infections were detected using broad-spectrum primers, enhancing the ability to amplify *Gamma*-PVs, followed by direct sequencing of PCR products, which may represent a limitation, as it could have led to the underestimation of some HPVs causing persistent infections and being present in lower viral loads. Therefore, it is possible that *Gamma*-PVs also cause transient infections of the anal canal, mostly having a commensal role and being transmitted through sexual and non-sexual routes.

## Conclusions

Based on results of our study and findings published previously, it could be concluded that *Gamma*-PVs are ubiquitous viruses with a wide tissue tropism, as they were detected in both mucosal and cutaneous sites, with the exception of members of the *Gamma*-6 species, which most probably only colonize mucosal ecological niches [21, 30, 53]. Our results provide new evidence that *Gamma*-PV infections of the anal canal may be associated with sexual behavior and the immune status of infected individuals. In conclusion, the present study expands the knowledge on *Gamma*-PVs’ tissue tropism, providing valuable data on the characteristics of HPV infection in the anal canal.

## Supplementary information

**Additional file 1: Table S1.** HPV types/putative types detected by *Gamma*-PV and CUT PCR assays. Novel putative HPV types are depicted in bold. Un: species not included in the current HPV taxonomy. *: The same HPV/putative type was found in different sets of samples. *Gamma*-X refers to sequences grouped in the genus *Gamma*-PV from which the prevailing HPV type/s and species could not be determined.

**Additional file 2: Table S2.** Distribution of HPV types in 21 initial and 23 follow-up anal canal swab samples from 21 individuals. Linear Array HPV Genotyping Test (Roche Diagnostics GmbH, Mannheim, Germany) is based on the reverse-line blot hybridization technique to detect the following 37 different mucosal HPV types distributed in 11 *Alpha*-PV species (high-risk HPV types depicted in bold): *Alpha*-1 (HPV42), *Alpha*-3 (HPV61, HPV62, HPV72, HPV81, HPV83, HPV84, CP6108), *Alpha*-5 (HPV26, **HPV51**, HPV69, HPV82, IS39), *Alpha*-6 (HPV53, **HPV56**, HPV66), *Alpha*-7 (**HPV18, HPV39, HPV45, HPV59, HPV68**, HPV70), *Alpha*-8 (HPV40), *Alpha*-9 (**HPV16, HPV31, HPV33, HPV35, HPV52, HPV58**, HPV67), *Alpha*-10 (HPV6, HPV11, HPV55), *Alpha*-11 (HPV73, HPV64), *Alpha*-13 (HPV54), *Alpha*-14 (HPV71). RHA Kit Skin (Beta) HPV assay (RHA; Diassay BV, Rijswijk, The Netherlands) detects the following 25 different *Beta*-PV types by reverse-line blot hybridization technique: *Beta*-1 (HPV5, HPV8, HPV12, HPV14, HPV19, HPV20, HPV21, HPV24, HPV25, HPV36, HPV47, HPV93), *Beta*-2 (HPV9, HPV15, HPV17, HPV22, HPV23, HPV37, HPV38, HPV80), *Beta*-3 (HPV49, HPV75, HPV76), *Beta*-4 (HPV92), *Beta*-5 (HPV96). HPV types (or negative results) identified simultaneously by more than one HPV test are depicted in bold. Samples containing possible unknown HPV type(s) are indicated as HPV-X. Pos: Positive; Neg: Negative; N/A: Not Analyzed.

**Additional file 3: Table S3.** Alignments of the forward and reverse primer sequences of CUT PCR, *Gamma*-PV PCR and CP PCR with corresponding regions of L1 and E1 ORFs of 64 selected *Gamma*-PV types. Lines and characters represent identical and mismatched nucleotides, respectively. Conserved sequences at the 3′ regions of *Gamma*-PV primers are highlighted in grey. Degenerate nucleotides of primers: i = inosine, y = c/t, r = a/g, w = a/t, h = a/c/t, m = a/c, s = g/c.

## Data Availability

The datasets used and/or analyzed during the current study are available from the corresponding author on reasonable request.
